# Heightened Fear Memory Retention During Infancy, but Not Number of Separation‐Induced Ultrasonic Vocalizations, Predicts a Fearful Phenotype in a Diverse Cohort of Rats

**DOI:** 10.1002/dev.70215

**Published:** 2026-11-03

**Authors:** Sylvia K. Harmon‐Jones, Rick Richardson

**Affiliations:** ^1^ School of Psychology, Faculty of Science, Medicine, and Health University of Wollongong Wollongong New South Wales Australia; ^2^ School of Psychology, Faculty of Science University of New South Wales Sydney New South Wales Australia

**Keywords:** anxiety, development, fear, individual differences, memory

## Abstract

Past work using male rats, unexposed to early life adversity, suggests that number of separation‐induced ultrasonic vocalizations (USVs) and retention of an aversive association during infancy predict later individual differences in fear behavior. The present study examined these associations using male and female rats that were either exposed or unexposed to early life adversity to test the generalizability of these findings. Separation‐induced USVs were measured early in infancy. Animals were then subjected to Pavlovian fear conditioning later in infancy, and individual differences in retention were measured 6 days later. In adulthood, individual differences in learned (contextual fear conditioning) and innate fear (light–dark box performance) were assessed. Exploratory factor analysis revealed that these adulthood measures loaded onto a single factor (fearfulness). Heightened memory retention of an aversive association during infancy positively predicted adulthood fearfulness, but number of USVs emitted was not a significant predictor. Neither sex nor exposure to early life adversity affected the size, nor direction, of these relations. These results suggest that heightened fear memory retention during infancy is a better predictor of adulthood fearfulness than separation‐induced USVs, and that these relationships generalize across sex and early life adversity exposure.

## 1 Introduction

Anxiety disorders are the most common of mental illnesses (Kessler et al. 2005) and are 10 times more costly to treat when they emerge early in development (Lee et al. 2014). As such, there is value in identifying early predictors of mental illness so that high‐risk individuals can be identified and targeted interventions can be administered (Kessler et al. 2007; Solmi et al. 2022). Because rodents reach maturity much more rapidly than humans and the behavioral response to fear/anxiety‐eliciting stimuli is largely conserved across species (Fox et al. 2005; e.g., both human and nonhuman animals often respond by freezing or avoiding the stimulus when they feel anxious/afraid), it may be beneficial to identify promising early predictors of anxiety‐like behavior using rodent models before attempting to translate findings to humans. This approach would allow promising early predictors of risk to be identified more rapidly than they would be if researchers relied solely on large‐scale, longitudinal studies in humans.

Although there may be advantages to identifying early predictors of anxiety using preclinical rodent models, there have been a number of failures to translate basic animal research to human samples (for reviews, see Garner 2014; Griebel and Holmes 2013). Some have proposed that these failures in translation may be partially accounted for by the strong focus on reducing individual differences and maintaining a large degree of experimental control in animal research (which is ironically often regarded as one of the major strengths of animal research; King et al. 2019). That is, clinical populations vary extensively on a number of factors (e.g., exposure to early life adversity, genetics, sex, medications, and diet), and animal researchers typically fail to model this heterogeneity in the lab. In the present study, we used a rodent model to examine early predictors of adulthood anxiety‐like behavior but introduced several factors that vary within clinical populations to better test the generalizability of the findings and identify individual differences that might moderate these relations.

In rodents, there have been two major approaches to studying anxiety‐like behavior: learned fear and innate fear. The learned fear approach employs Pavlovian fear conditioning procedures where animals receive pairings of a neutral conditioned stimulus (CS) with an aversive unconditioned stimulus (US). After repeated pairings of the CS and US, animals exhibit fear of the CS due to its association with the aversive US. Rodents that acquire greater fear of the CS are sometimes considered more anxious (Bush et al. 2007; Graham and Richardson 2016). A second approach has been to study *innate*, or unlearned, fear. This approach capitalizes on the conflict between rodents’ innate fear of bright, open, novel spaces and their natural motivation to explore novel environments (Ganella and Kim 2014). Rats that overcome their innate fear of novelty and explore these environments are considered to be less anxious.

### 1.1 Separation‐Induced Ultrasonic Vocalizations (USVs) as a Predictor of Adulthood Anxiety‐Like Behavior

Separation‐induced USVs have been identified as a potential predictor of adulthood anxiety‐like behavior in rats. Infant rodents emit USVs when separated from their mother which can encourage the mother to retrieve her offspring (Allin and Banks 1972; Wöhr and Schwarting 2008). Separation‐induced USVs are sometimes referred to as “distress calls” (Moles et al. 2004) or conceptualized as being indicative of “neediness” (Ragan et al. 2016; Harmon‐Jones and Richardson 2021). Brunelli (2005) hypothesized that separation‐induced USVs would predict adulthood anxiety‐like behavior based on Kagan and Snidman's (1991) research showing that children who cry more in response to novelty at 4 months of age are more likely to later exhibit behavioral inhibition (a phenotype associated with increased risk of developing anxiety disorders in adolescence/adulthood; Gladstone et al. 2005; Hayward et al. 1998; Schwartz et al. 1999). To test this hypothesis, Brunelli (2005) selectively bred rats based on the number of separation‐induced USVs they emitted during infancy, resulting in high‐USV and low‐USV lines. In adulthood, the high‐USV line demonstrated heightened innate fear (Dichter et al. 1996; Zimmerberg et al. 2005). Since rodents selectively bred to emit a high number of USVs during infancy show heightened innate fear in adulthood, emitting a greater number of separation‐induced USVs may be an early marker of an anxious phenotype in outbred (i.e., nonselectively bred) rats.

However, research using outbred rats has revealed an inconsistent relationship between number of separation‐induced USVs emitted during infancy and later fear behavior. In support of the hypothesis that separation‐induced USVs may be an early marker of an anxious phenotype in outbred rats, one study found that infant rats that exhibited better memory of a learned fearful association also emitted a greater number of separation‐induced USVs earlier in infancy (Harmon‐Jones et al. 2020). However, later work failed to replicate this relationship (Harmon‐Jones and Richardson 2021). Further, other research has demonstrated that emitting a greater number of separation‐induced USVs during infancy predicts lower innate fear in adulthood (Cordeiro et al. 2024; Wöhr and Schwarting 2008). Taken together, the relationship between separation‐induced USVs and adulthood fear behavior is unclear.

### 1.2 Fear Memory Retention During Infancy as a Predictor of Adulthood Anxiety‐Like Behavior

Various studies have shown that early life adversity is associated with accelerated maturation across a number of domains (for reviews, see Belsky 2019; Callaghan and Tottenham 2016; McLaughlin et al. 2019), including the development of long‐term fear memory retention (Callaghan and Richardson 2012; Cowan et al. 2013; Peng et al. 2019). Infant rodents typically forget learned CS–US associations quite rapidly relative to adults (Akers et al. 2012), a phenomenon known as *infantile amnesia*. In contrast, infants that have previously experienced repeated maternal separations (a model of early life adversity) show memory of the CS–US association for up to 30 days postconditioning (Callaghan and Richardson 2012).

Theoretical accounts, like the Stress Acceleration Hypothesis, propose that while accelerated maturation following early life adversity may be an adaptive response to harsh environments where caregivers are inconsistent or abusive, the emotion regulation difficulties commonly observed in those exposed to early life adversity may be a consequence of accelerated maturation of the neural circuitry underlying fear learning and emotion regulation (Callaghan and Tottenham 2016). For example, a number of studies show that exposure to early life adversity is associated with precocious structural (Lupien et al. 2011; Ono et al. 2008; Tottenham et al. 2010; Whittle et al. 2013) and functional (Gee et al. 2013) maturation of the amygdala and prefrontal cortex (PFC), which are critically involved in fear learning and emotion regulation across species. Callaghan and Tottenham (2016) proposed that this earlier transition to an “adult‐like” state may reduce developmental plasticity, which is disadvantageous for long‐term brain maturation and emotion regulation development.

While the above theoretical work draws primarily on between‐group comparisons between individuals exposed and those unexposed to early life adversity, we have previously examined whether individual differences in memory development during infancy predict later learned and innate fear in rats (Harmon‐Jones et al. 2020; Harmon‐Jones et al. 2021). In these studies, infant rats were trained to associate a white noise CS with an aversive footshock US. Memory of the association (i.e., fear of the white noise CS) was assessed ∼1 week later, a delay where the animals were expected to show individual differences in their memory of the association. Rodents that demonstrated better retention of the association (i.e., higher CS‐elicited freezing) exhibited heightened learned fear (Harmon‐Jones et al. 2020; Harmon‐Jones et al. 2021) and innate fear (Harmon‐Jones et al. 2020) in adulthood. Furthermore, rats that exhibited better retention of the CS–US association also emitted a greater number of separation‐induced USVs earlier in infancy (Harmon‐Jones et al. 2020; but see Harmon‐Jones and Richardson 2021). Taken together, these results suggest that enhanced retention of aversive associations is an early marker of increased innate and learned fear in adulthood, and that there may be overlap between individual differences in retention of an aversive association during infancy and separation‐induced USVs (i.e., that individual differences on these two measures may be a result of the same underlying phenotype).

In the present study, we aimed to replicate the finding that better retention of an aversive association memory retention during infancy predicts heightened learned and innate fear (as reported in Harmon‐Jones et al. 2020; Harmon‐Jones et al. 2021) across a relatively large sample of rats, and determine whether individual differences in separation‐induced USVs predict adulthood learned and innate fear among the same cohort of outbred rats. In addition, we examined the amount of unique variance that each of these early predictors explained in adulthood learned and innate fear. A difference in the amount of unique variance explained by each predictor may suggest that one of these measures is a better, or more effective, predictor of later learned and/or innate fear.

We also examined whether the relationships between separation‐induced USVs in early infancy, memory for an aversive association in later infancy, learned fear in adulthood, and innate fear in adulthood were affected by the sex of the animal or its exposure to early life adversity. Given that women are nearly twice as likely to develop an anxiety disorder compared to men (McLean et al. 2011), and estimates suggest that roughly 45% of all childhood‐onset disorders and up to 32% of all adult‐onset disorders are associated with exposure to early life adversity (Green et al. 2010), it is particularly important to develop methods of identifying low‐ and high‐risk individuals within these populations.

Similar to humans, sex and early life adversity exposure affect learned and innate fear in rodents. For example, rodent studies often report that animals exposed to an early life stressor (e.g., repeated separations from their mother in early infancy) exhibit heightened learned and innate fear later in life (Jin et al. 2018; Melo et al. 2018; Pryce et al. 2005). In addition, sex differences in learned and innate fear are often observed in rodents (e.g., Emtyazi et al. 2022; Gruene et al. 2015; Scholl et al. 2019). Contrary to human research, sex comparisons often demonstrate that female rodents show less learned and innate fear than males (Archer 1975; Imhof et al. 1993; Johnston and File 1991). Although the sex differences in learned and innate fear observed in rodent samples are opposite in direction to those found in humans (potential reasons for this discrepancy are addressed in depth in Section 4), similar sex‐specific mechanisms seem to mediate variations in female learned and innate fear across species (Li and Graham 2017; Mueller et al. 2014). Thus, there is still likely translational value in using female rodents to examine potential predictors of and treatments for anxiety.

Here, we employed a longitudinal design where infant rats were randomly assigned to maternal separation (i.e., exposure to early life adversity) or standard rearing (i.e., no exposure to early life adversity). Animals that were maternally separated were separated from their mothers for 3 h a day from postnatal day (P)2 to P14 (i.e., from the neonatal period to mid‐infancy) as per Callaghan and Richardson (2012). All of the subsequent procedures were adapted from those used in Harmon‐Jones et al. (2020). On a single day between P8 and P10 (i.e., during early infancy), each rat was briefly separated from their mother and the number of USVs emitted by each rat pup was measured. On either P17 or P18 (i.e., during late infancy), animals were subjected to Pavlovian fear conditioning and were tested 6 days later for memory of the aversive association (i.e., freezing in response to the CS). The animals were left undisturbed until adulthood, at which point innate fear was assessed using the light–dark box task. They were then subjected to contextual fear conditioning, tested for innate fear on the light–dark box again (to examine individual differences in innate fear following exposure to a stressor; i.e., the footshock used during contextual fear conditioning), and finally tested for contextually‐elicited fear (to examine individual differences in learned fear). See Figure 1 for a schematic of these procedures.

**FIGURE 1:**
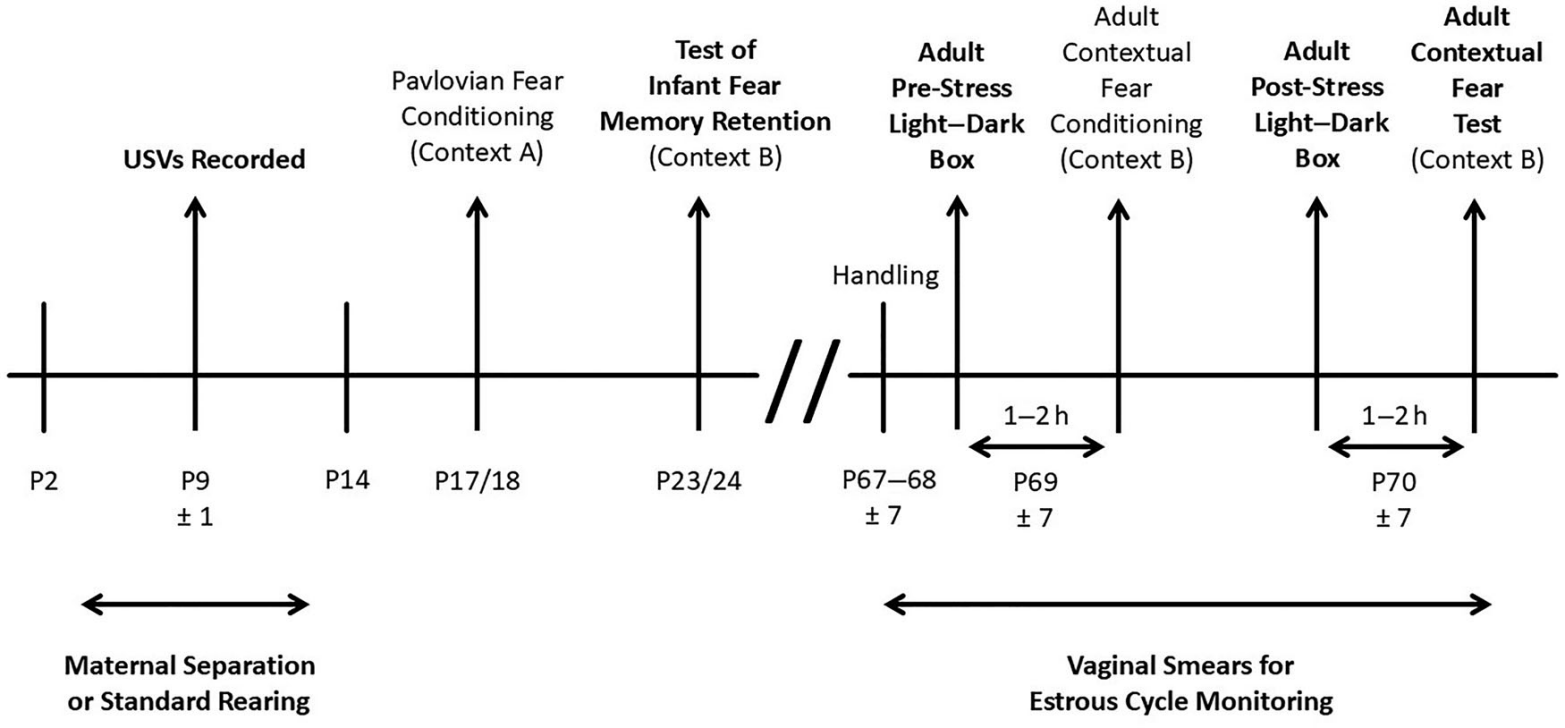
Schematic of the procedures. Measures in bold font were included in the present statistical analyses. P refers to postnatal day.

We had two primary, preregistered (https://osf.io/7yuwv/overview) research questions. First, we aimed to determine whether the relationship between memory retention for an aversive association in infancy and adult fear behavior, previously reported in male, standard‐reared rats (Harmon‐Jones et al. 2020), generalizes to female rats and maternally separated rats. Our preregistered hypothesis was that the relationship between memory retention for an aversive association in infancy and adulthood fear behavior would generalize to female rats and maternally separated animals (i.e., that neither sex nor rearing history would moderate the relationships). Second, we aimed to determine whether there is a difference in the predictive value of separation‐induced USVs and retention of an aversive association during infancy for measures of learned and innate fear in adulthood (i.e., does one of these early measures explain more variance in adulthood fear). As noted in the preregistration, this question was exploratory, and we did not have any a priori hypotheses regarding whether separation‐induced USVs or memory of an aversive association in infancy would be a stronger predictor of adulthood fear. After preregistering our hypotheses, design, and analysis plan, we decided to also use factor analysis to assess whether the variables measured across infancy and adulthood loaded onto the same factor (e.g., a fearful phenotype) based on factor analyses conducted on large samples of outbred rats that suggest that learned and innate fear measures are strongly related to one another (López‐Aumatell et al. 2009) and may be tapping into the same underlying construct (Aguilar et al. 2002). This factor analysis was exploratory and was not part of our preregistered analysis plan.

## 2 Methods

### 2.1 Transparency and Openness Statement

As described above, we preregistered our hypotheses, methods, and analyses on the Open Science Foundation's (OSF) website (https://osf.io/7yuwv/overview). There were only two deviations from our preregistration. First, we stated in our preregistration that we would analyze basal corticosterone levels using fecal samples. Although we collected fecal samples during handling, we did not analyze the samples. Second, we conducted an additional exploratory factor analysis and follow‐up analyses (described in the Section 2.6 and Section 3). All data used in our analyses are publicly available on our OSF project (https://osf.io/jmvfx/overview).

### 2.2 Data Stopping Rule

As described in our preregistration (https://osf.io/7yuwv/overview), we planned to run 30 animals per group (total *N* = 120) because 30 litters were undergoing maternal separation procedures in our lab for a different research project that was ongoing at the time of data collection. During maternal separation, the entire litter must be put through the experimental procedures, and only one male and one female per litter were used in the present experiment to avoid “litter effects” (Cowan and Richardson 2018).

### 2.3 Subjects

One hundred and nineteen experimentally naïve male (*n* = 59) and female (*n* = 60) Sprague–Dawley rats were obtained from the breeding colony in the School of Psychology at The University of New South Wales (UNSW). The animals were housed with their mother and littermates in opaque plastic boxes with wire tops (60 cm long × 30 cm wide × 27.5 cm high) from the first day of life (P0) until weaning, which took place after the test of memory retention of an aversive association on P23–24. Litters were culled to groups of eight on P2. Following weaning, animals were housed in same‐sex groups of two to four. All animals were kept on a 12‐h light/dark cycle (lights on at 7:00 a.m.) and were provided with food and water ad libitum. All procedures were approved by the UNSW animal care and ethics committee and conducted in line with the Australian code for the care and use of animals for scientific purposes (2013).

### 2.4 Procedure

#### 2.4.1 Maternal Separation

One group of animals (*n* = 59; 29 males, 30 females) was subjected to maternal separation from P2 to 14. Note that one litter was sexed incorrectly prior to being culled. As a result, 29, rather than 30, males were included in this study. Animals in this condition were removed from their home cage (between 9:00 a.m. and 12:00 p.m.), weighed, and placed in an incubator with their littermates for 3 h per day. The temperature of the incubator was kept between 27°C and 30°C. Roughly 3 cm of bedding was placed in the incubator so that the rats could burrow for extra warmth if needed. Standard‐reared animals (*n* = 60; 30 males, 30 females) were left undisturbed with their mother and littermates in their home cage during this time, and only received brief handling once a week during routine cage cleaning.

#### 2.4.2 USV Recording

As in Harmon‐Jones et al. (2020), on a single day between P8 and P10, each experimental animal was removed from its mother and littermates and placed into a rectangular Perspex chamber (30 cm long × 30 cm wide × 23 cm high) with a Perspex floor for 3 min. Separation‐induced USVs were measured using a Noldus Ultravox microphone, suspended from the ceiling of the chamber. Noldus Ultravox software was used to later count the number of USVs emitted by each rat within the range of 30–65 kHz (Portfors 2007).

#### 2.4.3 Pavlovian Fear Conditioning During Infancy

On either P17 or P18, each rat was placed into a chamber (13.5 cm long × 9 cm wide × 9 cm high) with flooring and two side walls made of stainless‐steel bars (2 mm diameter). The front wall, back wall, and ceiling were made of clear Perspex. This chamber will henceforth be referred to as Context A. An infrared camera was used to record the rats’ behavior. Infrared lighting was the only light source in Context A. There were two identical chambers that were used as Context A so that two animals were conditioned simultaneously. Each chamber was housed in a separate wood cabinet (in the same testing room), and each wood cabinet was fitted with a fan to muffle noise outside the chamber.

After being placed into Context A, no stimuli were presented for 2 min (a baseline period). Following the 2 min baseline period, animals were presented with six pairings of a white noise CS (8 dB above background noise, 10 s duration) and a mild footshock US (0.6 mA, 1 s duration) that co‐terminated with the CS. The footshock was administered through the grid flooring of the chamber. The intertrial interval (ITI) varied from 80 to 135 s (*M* = 110 s). The chamber was cleaned with water between conditioning sessions.

#### 2.4.4 Test of Fear Memory Retention in Infancy

Six days after conditioning (on either P23 or P24), each rat was placed individually into a novel context to test their memory of the CS–US association. The novel context (30 cm long × 30 cm wide × 23 cm high; henceforth referred to as Context B) differed from Context A in terms of size, illumination, and appearance. The ceiling and walls of Context B were made of clear Perspex. Two of the side walls were also covered in paper with vertical black and white stripes (5 cm wide). Stainless steel bars were used as flooring (1 mm diameter). An infrared camera was used to record the rats’ behavior, and a white LED was used to provide an additional light source. There was only one chamber that was used as Context B, housed in a wood cabinet that was fitted with a fan to muffle noise outside the chamber. All animals were tested individually.

After a 1‐min baseline period (where no stimuli were presented), the CS was presented continuously for 2 min. Context B was cleaned with water between testing sessions.

#### 2.4.5 Handling and Vaginal Smears

In adulthood, all animals were handled for 2 days for 3–5 min per day (starting on P60–74). Commencing on the first day of handling, female rats underwent vaginal smears daily to determine estrous phase. A cotton swab was dipped in 0.9% saline and then inserted into the rat's vaginal canal and twisted to collect epithelial cells. The cotton swab was then rubbed over a microscope slide to transfer the epithelial cells. Once dry, the slides were dyed using KwikDiff Stain Kit (Thermofisher, Australia) and examined under a light microscope (x10). The presence of leukocytes, nucleated cells, and cornified cells was examined to determine which phase of the estrous cycle (estrus, metestrus, diestrus, or proestrus) each female rat was in on each day of testing, as described in Becker et al. (2005).

#### 2.4.6 Pre‐Stress Light–Dark Box in Adulthood

The day after handling, animals were tested for innate fear in the light–dark box: a rectangular chamber (38 cm long × 18 cm wide × 20 cm high), separated into two 18.5‐cm‐long compartments by a wall in the center. The dark chamber was made of opaque black Perspex. In contrast, the light chamber had white Perspex walls and a transparent Perspex lid to allow in ambient light. Animals were able to move between the two compartments via an opening in the central wall (7.5 cm wide × 7.5 cm high) that could also be closed with a painted metal sliding door. A video camera was suspended above the light–dark box to record the rats’ behavior.

Each animal was placed into the dark side of the chamber and confined there for 1 min. After 1 min, the sliding metal door was removed so that the animal could move between the dark and light chambers for 5 min. The apparatus was cleaned with water between each testing session.

#### 2.4.7 Contextual Fear Conditioning in Adulthood

One to two hours after testing on the light–dark box, each rat was placed in Context B for 2 min prior to receiving a single 0.6 mA footshock (1 s duration). Context B was as described above, except that a larger stainless‐steel grid (bars 3 mm diameter; 1.5 cm apart) was used for the floor. The context was cleaned with water between each conditioning session.

#### 2.4.8 Post‐Stress Light–Dark Box in Adulthood

Because past research demonstrated that differences in innate fear observed between rats that expressed better/poorer fear memory retention during infancy are potentiated by exposure to a footshock stressor (Harmon‐Jones et al. 2020), we conducted a second light–dark box test the day after contextual fear conditioning. We hypothesized that the shock presented during the contextual fear conditioning procedure would act as a stressor, and as such, innate fear would be higher at this second test, particularly for animals that had better fear memory retention during infancy. This test session was conducted in the same manner as the pre‐stress light–dark box.

#### 2.4.9 Contextual Fear Test in Adulthood

One to two hours after the post‐stress light–dark box test, the rats were returned to Context B for 3 min to assess individual differences in contextual fear learning/memory. No other stimuli were presented during this test.

### 2.5 Scoring

#### 2.5.1 Freezing

Freezing was used as a measure of learned fear during the test of CS‐elicited fear during infancy and the contextual fear test in adulthood. Freezing is defined as the absence of all movement aside from that necessary for respiration (Fanselow 1980), and it was measured using a time‐sampling procedure where each rat was judged as either freezing or not freezing every 3 s. The number of observations where the rat was judged as “freezing” was divided by the total number of observations to give a measure of the percent of time each rat spent freezing.

A second observer, who was blind to condition, cross‐scored ∼30% of the test data. Interobserver reliability was very high for both the test of conditioned fear in infancy (*r*_31_ = 0.97, *p* < 0.001) and the test of contextual fear in adulthood (*r*_34_ = 0.97, *p* < 0.001).

#### 2.5.2 Light–Dark Box Performance

Three measures of innate fear were used to assess individual differences in behavior in the light–dark box. The first measure was latency, defined as the time in seconds that it took the rat to enter the light compartment. Longer latencies are indicative of *higher* innate fear. The second measure was time, defined as the total amount of time, in seconds, that a rat spent in the light compartment. Spending more time in the light compartment is indicative of *lower* innate fear. The third measure was entries, defined as the number of times that a rat entered the light compartment. A greater number of entries is indicative of *lower* innate fear. In order for an entry to be scored, the rat needed to have placed all four paws in the light compartment. Similarly, for an exit to be scored, the rat must have placed all four paws in the dark compartment.

A second observer, who was blind to condition, cross‐scored ∼30% of the test data. Interobserver reliability was very high across all measures on both the pre‐stress test (latency: *r*_33_ = 1.00, *p* < 0.001; time: *r*_33_ = 0.99, *p* < 0.001; entries: *r*_33_ = 1.00, *p* < 0.001) and post‐stress test (latency: *r*_32_ = 1.00, *p* < 0.001; time: *r*_32_ = 1.00, *p* < 0.001; entries: *r*_32_ = 0.99, *p* < 0.001).

### 2.6 Analysis

#### 2.6.1 Preregistered Analyses

The following analyses were preregistered (https://osf.io/7yuwv/overview) and were conducted using IBM SPSS 27.

Because the three measures typically taken on the light–dark box task (i.e., latency to enter, time spent on the light side, and number of entries into the light side) are all intended to measure the same underlying construct, we chose to create a composite measure of innate fear behavior. Latency was first reverse‐scored (i.e., each animal's score was subtracted from the maximum score of 300 s) so that *higher* scores on all measures represented *heightened* exploratory behavior. Each measure was then standardized (i.e., *z*‐scored) so that all measures were in the same units. The mean of the three standardized scores was then taken and used in all subsequent analyses to simplify our analyses and to provide an aggregate estimate of exploratory behavior. Note that increased exploratory behavior is indicative of reduced innate fear on this task.

We first assessed for the presence of outliers (defined as a *z*‐score >|3.29|; Tabachnick and Fiddell 2019) in all measured variables, and no data points met the criteria. We then compared performance on all adult measures (i.e., context‐elicited fear and light–dark box performance) between female rats in proestrus (a phase of the estrous cycle associated with high levels of estrogen) and female rats that were not in proestrus (i.e., estrus, diestrus, or metestrus) using an independent samples *t*‐test based on evidence that females in proestrus exhibit lower levels of innate fear (Frye et al. 2000; Frye and Walf 2002; Marcondes et al. 2001). Between‐subjects factorial ANOVAs were then used to determine whether there was an effect of sex, early life adversity exposure, or an interaction between sex and early life adversity exposure on any of the dependent measures.

The within‐cell correlations were then calculated for all outcome measures using Pearson's *r*. Fisher's *r*‐to‐*z* transformation was used to determine if there were any significant differences in the size/direction of the correlations between groups. If no significant differences were detected, the correlations were collapsed using the mini meta‐analytic strategy described by Goh et al. (2016) to increase statistical power and obtain an overall weighted *r* value. Meta‐analyzed correlations were then compared using the approach recommended by Borenstein et al. (2009). The mean difference between the correlations was divided by the standard error of the difference to calculate a *z*‐score. If no significant differences were observed, the correlations were collapsed across sex.

Finally, linear regressions were conducted for each adulthood outcome measure. Number of separation‐induced USVs emitted during infancy, memory retention of an aversive association later in infancy, and the interaction between these two variables were entered as predictors. Here, we were interested in whether (a) USVs or memory retention of an aversive association during infancy predicted more unique variance in the adulthood outcome measures and (b) whether the relation between memory of an aversive association and the outcome measures depended upon number of USVs emitted (e.g., were animals that emitted a high number of USVs *and* expressed heightened memory of an aversive association at higher risk than animals that emitted a high number of USVs but expressed reduced memory retention?). Number of USVs emitted and performance on the test of memory of an aversive association during infancy were both mean‐centered prior to inclusion in the models. Any other variables that had a significant effect on the outcome measure in the between‐subjects factorial ANOVA or independent samples *t*‐test were also included in the regression as covariates.

#### 2.6.2 Exploratory Analyses

After conducting the preregistered analyses, based on previous factor analyses that suggest that learned and innate fear measures are strongly related to one another (López‐Aumatell et al. 2009), we examined whether the medium‐to‐large correlations between measures (see Section 3) suggested that these tasks were tapping into the same underlying factor (as suggested by Aguilar et al. 2002). As such, we subjected all measured variables (i.e., USVs emitted, memory retention of an aversive association during infancy, pre‐ and post‐stress performance on the light–dark box, and context‐elicited fear) to an exploratory factor analysis.

The factor analysis revealed that the adulthood measures (but not the infant measures) loaded on the same factor. As such, we created a composite measure of adulthood fearfulness. We then entered number of USVs emitted, memory of an aversive association during infancy, and their interaction into a linear regression to determine if these variables significantly predicted this overall adult behavioral phenotype.

#### 2.6.3 Missing Data

One maternally separated male animal died before testing in adulthood. This animal was excluded from all analyses. USV data were missing for three animals (one maternally separated male and two maternally separated females) due to experimenter error (final *n*s for USVs: standard‐reared males = 30; standard‐reared females = 30; maternally separated males = 27; maternally separated females = 28). Data were missing for the test of memory retention of an aversive association during infancy for five male, maternally separated animals because we initially used a longer delay (14 days) between fear conditioning and testing based on past work demonstrating that maternally separated male rats retain conditioned associations longer than standard‐reared males (Callaghan and Richardson 2012; Cowan et al. 2013; Peng et al. 2019). However, after testing these five males, it became clear that by 14 days, fear memory retention was very low on average (*M* = 4%, SD = 3.79%). As such, we chose to use a 6‐day delay between fear conditioning and testing for all remaining animals so that we could detect variability in fear memory retention among all groups (final *n*s for memory of an aversive association during infancy: standard‐reared males = 30; standard‐reared females = 30; maternally separated males = 23; maternally separated females = 30). Post‐stressor light–dark box data were missing for two maternally separated female animals because these animals did not receive the shock at contextual fear conditioning due to equipment failure, meaning that they had not received the stressor (final *n*s for post‐stressor light–dark box: standard‐reared males = 30; standard‐reared females = 30; maternally separated males = 28; maternally separated females = 28). These two animals and one standard‐reared male animal (due to experimenter error) also were not tested for contextual fear memory (final *n*s for contextual fear memory: standard‐reared males = 29; standard‐reared females = 30; maternally separated males = 28; maternally separated females = 28).

## 3 Results

### 3.1 Adulthood Performance—Effects of Proestrus

There was no evidence of an effect of being in proestrus at the time of testing on any adulthood measure (see the Supporting Information). As such, estrous phase was not included as a covariate in any subsequent analyses.

### 3.2 Effects of Early Life Adversity

There was a significant effect of rearing on number of USVs in infancy (*F*_1, 111_ = 6.17, *p* = 0.015; *η*_p_^2^ = 0.05) where maternally separated animals emitted more USVs than standard‐reared animals. See Table 1 for all descriptive statistics. There were no significant effects of rearing or interactions between rearing and sex on any other outcome measure (see the Supporting Information).

**TABLE 1:** Means and standard deviations for each measured variable by sex and early life adversity exposure, and across the entire sample.

	Infant number of USVs		Infant % CS‐elicited freezing		Adult pre‐stress light‐dark box		Adult post‐stress light‐dark box		Adult % context‐elicited freezing		Overall adult fearfulness	
	*M*	SD	*M*	SD	*M*	SD	*M*	SD	*M*	SD	*M*	SD
Males	459.58	185.76	40.24	28.54	−0.43	0.85	−0.43	0.47	55.96	26.52	0.37	0.60
Females	467.22	190.55	45.21	27.13	0.42	0.78	0.43	1.08	41.75	26.02	−0.38	0.75
Maternally separated	507.96	195.38	39.39	27.31	−0.04	0.94	0.09	1.01	44.61	29.44	−0.07	0.83
Males	517.19	195.51	35.11	28.66	−0.57	0.89	−0.47	0.38	52.08	26.67	0.39	0.59
Females	499.07	198.42	42.67	26.24	0.46	0.67	0.65	1.12	37.14	30.64	−0.54	0.79
Standard‐reared	422.62	171.40	45.96	28.07	0.04	0.90	−0.08	0.87	52.77	24.28	0.05	0.72
Males	407.73	162.77	44.17	28.30	−0.30	0.79	−0.40	0.54	59.71	26.29	0.35	0.62
Females	437.50	181.15	47.75	28.20	0.37	0.89	0.23	1.01	46.06	20.42	−0.24	0.69
Overall	463.43	187.40	42.88	27.79	0.00	0.91	0.00	0.94	48.80	27.11	−0.01	0.78

*Note: M* refers to mean. SD refers to standard deviation. USVs refer to ultrasonic vocalizations. The means and SDs presented for “Adult pre‐stress light‐dark box” and “Adult post‐stress light‐dark box” refer to the composite measures of light‐dark box exploration from the pre‐stress and post‐stress tests, as these variables were used in all subsequent analyses.

### 3.3 Effects of Sex

There were main effects of sex on pre‐stress (*F*_1, 114_ = 32.26, *p* < 0.001, *η*_p_^2^ = 0.221) and post‐stress (*F*_1, 112_ = 32.76, *p* < 0.001, *η*_p_^2^ = 0.23) light‐dark box performance, where females exhibited more exploratory behavior (i.e., less innate fear) than males. There was also main effect of sex (*F*_1, 111_ = 8.58, *p* = 0.004, *η*_p_^2^ = 0.07) where females had lower context‐elicited freezing (*M* = 41.75, SD = 26.02) than males (*M* = 55.96, SD = 26.52). However, there were no effects of sex on number of USVs or fear memory retention during infancy (see the Supporting Information).

### 3.4 Bivariate Correlations Between Measured Variables

As per our preregistered analysis plan, we assessed whether there were differences in the size/direction of the relations between the measured variables (i.e., number of USVs, CS‐elicited freezing during infancy, pre‐ and post‐stress light–dark box performance in adulthood, and context‐elicited freezing in adulthood) between standard‐reared and maternally separated animals of the same sex. There were no significant differences in the size/direction of the correlations (largest *Z* = 1.62, *p* = 0.105). As such, the correlations were meta‐analyzed within sex (see Tables S1 and S2 for the correlations between measured variables for male and female animals). There was also no evidence of a significant sex difference in the magnitude of these relations (largest *Z* = 1.69, *p* = 0.092). As such, the correlations were next meta‐analyzed across sex (see Table 2).

**TABLE 2:** Bivariate correlations between measured variables across the entire sample.

	Infant USVs	Infant CS‐elicited freezing	Adult pre‐stress light‐dark box	Adult post‐stress light‐dark box
Infant CS‐elicited freezing	−0.16			
Adult pre‐stress light‐dark box	0.13	−0.15		
Adult post‐stress light‐dark box	0.16	−0.15	0.45^***^	
Adult context‐elicited freezing	−0.02	0.28^**^	−0.37^***^	−0.48^***^

*Note:* USVs refer to ultrasonic vocalizations. “Adult pre‐stress light‐dark box” and “Adult post‐stress light‐dark box” refer to the composite measures of light‐dark box exploration from the pre‐stress and post‐stress tests.

^*^ *p* < 0.050; ^**^ *p* < 0.010; ^***^ *p* < 0.001.

In line with predictions, better retention of an aversive association during infancy (i.e., higher CS‐elicited freezing) positively predicted context‐elicited freezing in adulthood. Although the correlations between retention of an aversive association during infancy and performance on the light–dark box were in the predicted direction (i.e., stronger memory retention predicted greater innate fear/lower exploration), these correlations did not reach statistical significance. The number of USVs emitted was not significantly correlated with any other measured variables.

Greater innate fear (i.e., lower exploration) on the pre‐stress light–dark box measure was associated with greater innate fear on the post‐stress measure and higher context‐elicited freezing. Similarly, greater innate fear on the post‐stress light–dark box measure was associated with greater context‐elicited freezing.

### 3.5 Linear Regressions Predicting Adulthood Outcome Measures

Number of USVs emitted, CS‐elicited freezing during infancy, and their interaction were simultaneously entered into separate linear regressions predicting each adulthood outcome measure (i.e., performance on the light–dark box both pre‐ and post‐stress, and the test of context‐elicited freezing). Sex and early life adversity exposure were also entered as predictors in the regressions, because the between‐subjects ANOVAs demonstrated that sex had a significant effect on all adulthood measures and that early life adversity exposure had a significant effect on number of USVs.

#### 3.5.1 Pre‐Stress Light–Dark Box

The overall model was significant with approximately 30% of the variance in performance accounted for (*R* = 0.54, *R* ^2^ = 0.30, *F*_5, 104_ = 8.70, *p* < 0.001). Females showed less innate fear (i.e., explored more) on this task than males. In addition, the interaction between USVs emitted and CS‐elicited freezing during infancy was significant. All other predictors were nonsignificant (see Table 3).

**TABLE 3:** Contributions of individual predictors to variance in adulthood outcome measures across sex and early life adversity exposure.

	Infant USVs		Infant CS‐elicited freezing		Infant USVs × Infant CS‐elicited freezing		Sex		Early life adversity exposure	
	*B*	*p*	*B*	*p*	*B*	*p*	*B*	*p*	*B*	*p*
Adult pre‐stress light‐dark box	0.09	0.281	−0.11	0.200	**0.19**	**0.023**	**0.46**	**<0.001**	−0.12	0.164
Adult post‐stress light‐dark box	0.13	0.137	−0.09	0.322	−0.03	0.712	**0.47**	**<0.001**	0.02	0.826
Adult context‐elicited freezing	0.03	0.731	**0.28**	**0.003**	−0.003	0.972	**−0.33**	**0.001**	−0.06	0.509

*Note:* USVs refer to ultrasonic vocalizations. *B* refers to the standardized beta coefficient. “Adult pre‐stress light‐dark box” and “Adult post‐stress light‐dark box” refer to the composite measures of light‐dark box exploration from the pre‐stress and post‐stress tests. Statistically significant predictors are in bold.

We used PROCESS (Hayes 2013) to decompose the interaction by examining the conditional effects of number of USVs emitted at ±1 SD from the mean of CS‐elicited freezing during infancy. If animals exhibited better fear memory retention (i.e., 1 SD above the mean), emitting more USVs during infancy was associated with lower innate fear on the light–dark box in adulthood (i.e., number of USVs was positively associated with exploration; *b* = 0.0015, *p* = 0.025, 95% CI [0.0002, 0.0028]). In contrast, number of USVs emitted did not significantly predict performance on the light–dark box for animals that exhibited poorer fear memory retention (i.e., 1 SD below the mean; *b* = −0.0007, *p* = 0.276, 95% CI [−0.0019, 0.0005]).

#### 3.5.2 Post‐Stress Light–Dark Box

The overall model was significant with approximately 25% of the variance in performance accounted for (*R* = 0.50, *R* ^2^ = 0.25, *F*_5, 102_ = 6.89, *p* < 0.001). The only significant predictor of performance on the post‐stress light–dark box was the sex of the animal (see Table 3), with females exhibiting less innate fear than males.

#### 3.5.3 Context‐Elicited Freezing

The overall model was significant with approximately 18% of the variance accounted for (*R* = 0.42, *R* ^2^ = 0.18, *F*_5, 101_ = 4.37, *p* = 0.001). Females exhibited lower context‐elicited freezing than males. In addition, as predicted, CS‐elicited freezing during infancy positively predicted context‐elicited freezing in adulthood. That is, animals that displayed better retention of an aversive association during infancy also demonstrated higher context‐elicited freezing in adulthood. All other predictors were nonsignificant (see Table 3).

### 3.6 Exploratory Factor Analysis

After conducting the preregistered analyses above, based on previous factor analyses that suggest that learned and innate fear measures are strongly related to one another (López‐Aumatell et al. 2009), we assessed whether the adulthood measures were tapping into a similar underlying construct (e.g., fearfulness; as suggested by Aguilar et al. [2002] and Flaherty et al. [1998]) because the bivariate correlations revealed moderate to large associations between the adulthood measures. We also examined whether the infant measures loaded onto a separate factor, suggesting that infant and adulthood behavior are distinct constructs (even though they may be correlated). These analyses were exploratory and were not part of our preregistered analysis plan.

All measured variables were subjected to factor analysis with principal axis factoring with oblique rotation (direct oblimin), because if multiple factors emerged, we suspected that they would be correlated. The Kaiser–Meyer–Olkin (KMO) measure of sampling adequacy (MSA) was 0.63, suggesting that the sample size was mediocre to middling (Kaiser and Rice 1974). While this may not be ideal, we continued with the analysis because this study included a relatively large number of animals compared to the average rodent study. Further, the individual MSAs were >0.49 for all variables, just under the acceptable limit of 0.50 (Kaiser 1974). Bartlett's test of sphericity was significant (*χ* ^2^_10_ = 94.90, *p* < 0.001), suggesting that there may be multicollinearity among the measured variables. However, all correlation coefficients were <0.57, which is well below the recommended limit of 0.80 (Field 2018), indicating that the measured variables did not show multicollinearity.

There were two factors with an eigenvalue over Kaiser's criterion of 1 (Kaiser 1960). The first factor had an eigenvalue of 2.12 and explained 42.45% of the variance. The second factor had an eigenvalue of 1.09 and explained 21.76% of the variance. However, the scree plot had a point of inflexion at the second component, indicating a one‐factor solution (Field 2009). Nonetheless, because some researchers recommend specifying too many factors rather than too few (Hayton et al. 2004; Zwick and Velicer 1986), we retained the two‐factor solution.

The pattern matrix was inspected, and measures that loaded <0.50 were removed (i.e., number of USVs; see Table S3 for the original loadings). No items were cross‐loaded.

Principal axis factoring with oblique rotation (direct oblimin) was repeated with number of USVs omitted from the factor analysis to ensure that the factor structure was retained. Upon re‐analysis, fear memory retention during infancy loaded <0.50 on Factor 2 (see Table S4). As such, a single‐factor solution seemed most appropriate. The analysis was repeated (without oblique rotation, as we expected a single‐factor solution), omitting CS‐elicited freezing during infancy. A single factor emerged with an eigenvalue of 2.00, explaining 66.79% of the variance. All measures had acceptable loadings (i.e., >0.50) on this factor (see Table 4). Given that all adult measures of learned and innate fear loaded sufficiently on this factor, we decided to refer to this factor as *Fearfulness*.

**TABLE 4:** Pattern matrix for the final single‐factor solution.

	Factor
	1
Adult pre‐stress light‐dark box	**0.665**
Adult post‐stress light‐dark box	**0.835**
Adult context‐elicited freezing	**−0.631**

*Note:* Bolded factor loadings indicate that the measure loaded acceptably (i.e., >0.50) on the factor. “Adult pre‐stress light‐dark box” and “Adult post‐stress light‐dark box” refer to the composite measures of light‐dark box exploration from the pre‐stress and post‐stress tests.

Because context‐elicited fear had a negative factor loading while pre‐ and post‐stress performance on the light–dark box had positive loadings, we reverse‐scored pre‐ and post‐stress performance on the light–dark box so that higher scores on all measures represented greater fearfulness. We then *z*‐scored context‐elicited fear (so that all variables were in the same units of measurement) and took the mean of the three adulthood measures to create a composite measure of Fearfulness.

### 3.7 Predicting Overall Fearfulness

Although the bivariate correlations and linear regressions from our preregistered analyses did not clearly support our hypotheses that number of USVs emitted and/or retention of an aversive association during infancy would reliably predict learned and innate fear in adulthood, we assessed whether retention of an aversive association during infancy and/or number of USVs emitted predicted an overall fearful phenotype in adulthood.

#### 3.7.1 Group Differences

We first confirmed that there was no effect of being in proestrus on the first (*t*_56_ = 0.02, *p* = 0.987, *d* = 0.01) or second day (*t*_56_ = 1.29, *p* = 0.202, *d* = 0.40) of testing on Fearfulness. We then used a between‐subjects ANOVA to assess for any effect of sex, early life adversity exposure, or their interaction on Fearfulness. There was a main effect of sex (*F*_1, 111_ = 35.90, *p* < 0.001, *η*_p_^2^ = 0.24) where males were more Fearful than females. There was no significant effect of early life adversity exposure (*F*_1, 111_ = 1.10, *p* = 0.297, *η*_p_^2^ = 0.01) nor interaction between sex and exposure to early life adversity (*F*_1, 111_ = 1.80, *p* = 0.183, *η*_p_^2^ = 0.02).

#### 3.7.2 Bivariate Correlations

We then compared the within‐cell correlations and found no significant differences in the size/magnitude of the correlations between Fearfulness and number of USVs emitted or retention of an aversive association during infancy for animals of the same sex that differed in terms of exposure to early life adversity (largest *Z* = 1.05, *p* = 0.147).

Once we collapsed across early life adversity exposure, there was no significant correlation between number of USVs emitted and Fearfulness for males (*r*_overall_ = −0.09, *p* = 0.518) nor females (*r*_overall_ = −0.17, *p* = 0.237). In line with predictions, there was a trend toward a correlation between better memory retention during infancy and heightened Fearfulness for both males (*r*_overall_ = 0.25, *p* = 0.083) and females (*r*_overall_ = 0.23, *p* = 0.089). However, these correlations did not reach traditional levels of statistical significance. There was no evidence of a significant sex difference for the size/direction of either correlation (largest *Z* = 0.38, *p* = 0.705). As such, the correlations were collapsed across sex. There was no significant correlation between number of USVs emitted and Fearfulness (*r*_overall_ = −0.13, *p* = 0.196). However, as predicted, better retention of an aversive association during infancy was significantly correlated with heightened Fearfulness in adulthood when collapsed across sex and exposure to early life adversity (*r*_overall_ = 0.24, *p* = 0.015).

#### 3.7.3 Linear Regression Predicting Overall Fearfulness

Number of USVs emitted during infancy, CS‐elicited freezing during infancy, and their interaction were entered simultaneously as predictors of our overall measure of adulthood Fearfulness. Sex of the animal and early life adversity exposure were also included as covariates, because the between‐subjects ANOVAs demonstrated that sex had a significant effect on Fearfulness and early life adversity exposure had a significant effect on number of USVs.

The overall model predicting Fearfulness was significant with approximately 31% of the variance accounted for (*R* = 0.55, *R* ^2^ = 0.31, *F*_5, 101_ = 8.96, *p* < 0.001). Males were more Fearful than females (*B* = −0.51, *p* < 0.001). In line with predictions, higher CS‐elicited freezing during infancy significantly predicted heightened adulthood Fearfulness (*B* = 0.19, *p* = 0.026). However, number of USVs emitted (*B* = −0.08, *p* = 0.392), the interaction between number of USVs and CS‐elicited freezing during infancy (*B* = −0.06, *p* = 0.476), and exposure to early life adversity (*B* = 0.01, *p* = 0.895) were nonsignificant predictors of adulthood Fearfulness.

## 4 Discussion

The results of the present study suggest that individual differences in retention of an aversive association during infancy predict variations in adulthood fear behavior. Neither the sex of the animal nor prior exposure to early life stress significantly affected the size/direction of this relation. Our analyses examining individual measures of learned and innate fear in adulthood provided mixed support for the hypothesis that exhibiting “better” retention of an aversive association during infancy would be associated with heightened fear behavior. However, the results of a factor analysis study suggest that measures of innate fear (i.e., the light–dark box) and learned fear (i.e., context‐conditioned fear) tap into a similar underlying construct (e.g., fearfulness). Once the adulthood measures of learned and innate fear were combined into a single factor, representing adulthood fearfulness, better retention of an aversive association during infancy was indeed predictive of adulthood fear behavior. Given that the present factor analysis (and previous factor analyses; Aguilar et al. 2002; Flaherty et al. 1998) suggests that measures of learned and innate fear are capturing the same latent variable, averaging across the measures of learned and innate fear likely resulted in a more reliable estimate of each animal's trait fearfulness in adulthood (Bollen 1989).

Although exhibiting enhanced retention of an aversive association during infancy has previously been shown to predict learned and innate fear (Harmon‐Jones et al. 2020; Harmon‐Jones et al. 2021), this past work only included male, standard‐reared rats. Given that being female (e.g., McLean et al. 2011) and/or being exposed to early life adversity (e.g., Green et al. 2010) are major risk factors for the development of an anxiety disorder, it is critical that researchers develop methods of identifying those at particularly high risk within these groups. Furthermore, calls have increasingly been made to include both male and female rodents in basic research in light of mounting evidence demonstrating that sex moderates various drug and experimental effects (e.g., as described in Clayton [2016] and Tannenbaum et al. [2016]). The present results extend past work by demonstrating that exhibiting better retention of an aversive association during infancy predicts an overall fearful phenotype across a diverse sample of rodents, including animals that represent high‐risk groups (i.e., female rodents and rodents exposed to early life adversity).

That said, we did not find any effect of maternal separation (i.e., a manipulation used to model early life adversity) on all measures, aside from number of USVs emitted during infancy. Maternal separation has previously been shown to cause enhanced retention of aversive associations during infancy (Callaghan and Richardson 2012; Cowan et al. 2013; Peng et al. 2019) and heightened anxiety‐like behavior in adulthood (Jin et al. 2018; Melo et al. 2018; Pryce et al. 2005), resulting in maternal separation commonly being used as a rodent analog of early life adversity exposure. A recent meta‐analysis found that maternal separation leads to a moderate increase in innate fear on the open‐field test and elevated plus maze in adult rats. However, once publication bias was controlled for in this meta‐analysis, the anxiogenic effect of maternal separation was no longer statistically significant (Wang et al. 2020). Furthermore, power analyses indicated that groups of 100 animals are necessary to obtain 0.8 power to detect a moderate effect of maternal separation, a sample size that is five to 10 times larger than that typically used in rodent research (Wang et al. 2020). Because the vast majority of rodent studies are highly underpowered to detect an effect of maternal separation, it is unclear whether the anxiogenic effect of maternal separation that is often referred to in the literature is a robust effect, an artifact of publication bias, and/or an effect inflated due to running and publishing underpowered studies. Given that we did not observe an anxiogenic effect of maternal separation in the present study, it is unclear whether individual differences in retention of an aversive association during infancy would still predict fearfulness in adulthood in a cohort of “high‐risk” animals (e.g., animals that show an anxiogenic effect of maternal separation or another model of early life adversity).

We also did not find a relation between individual differences in separation‐induced USVs and adulthood fear behavior. Past work has shown that rats selectively bred to exhibit high levels of separation‐induced USVs express heightened innate fear in adulthood (Brunelli 2005; Zimmerberg et al. 2005). In addition, natural variations in separation‐induced USVs have been shown to positively predict retention of an aversive association later in infancy (Harmon‐Jones et al. 2020). However, as noted in Section 1, this relationship has failed to replicate (Harmon‐Jones and Richardson 2021), and other work has shown a negative relationship between USV emission during infancy and innate fear in adulthood (Cordeiro et al. 2024; Wöhr and Schwarting 2008). Wöhr and Schwarting (2008) suggested that emitting separation‐induced USVs may be an active coping strategy, as infants that emit USVs should encourage their mother to retrieve them. Thus, emitting a higher number of USVs during infancy could be indicative of resilience. Relatedly, in the present study, male animals that emitted a greater number of separation‐induced USVs showed “poorer” retention of an aversive association later in infancy. Additionally, we found an interaction between the number of separation‐induced USVs animals emitted and their retention of an aversive association in infancy. Specifically, when animals expressed heightened retention of an aversive association during infancy *and* emitted a greater number of separation‐induced USVs, they then showed lower innate fear on the pre‐stress light–dark box in adulthood. As such, more work is needed to clarify whether individual differences in USV emission during infancy are indicative of a fearful phenotype, a resilient phenotype, or something entirely different (e.g., cold sensitivity; Allin and Banks 1971; Blumberg and Alberts 1990).

### 4.1 Limitations

The adulthood measures of learned and innate fear all used reduced locomotion as a proxy for fear behavior. Using reduced locomotion as a proxy for fear behavior could pose a problem for examining sex differences in fear behavior because females are more active than males (e.g., Bernatova et al. 2010; McCormick et al. 2005; Ramos et al. 1997) and some have proposed that these measures are not appropriate to measure innate fear in female rats (Börchers et al. 2022; Fernandes et al. 1999; Shansky 2018; but see Doremus et al. 2006).

In addition, the fact that all of the adulthood measures assessed individual differences in locomotion may make it difficult to interpret the results of our factor analysis. That is, rather than these measures tapping into an underlying construct of fearfulness, they may simply tap into an underlying construct of activity. However, previous factor analyses have generally demonstrated that locomotion loads on a separate factor from innate fear measures (at least for males; Arrant et al. 2013; Fernandes et al. 1999), somewhat mitigating this concern.

We also used the same context (Context B) to test memory of the CS in infancy and condition contextual fear in adulthood, and we cannot rule out the possibility that animals had some memory of the context in adulthood due to prior exposure. However, because the footshock US was never presented in the test context during infancy, it seems unlikely that animals would have learned to fear the test context. While it is theoretically possible that second‐order conditioning could have occurred during the infant test whereby animals learned to fear the test context due to presentation of the white noise CS (Holmes et al. 2022), it would be highly unlikely that this relatively weak association would be remembered ∼40 days later when the adult context conditioning procedure occurred. Nonetheless, future experiments should disentangle whether infants remember test contexts in adulthood and, if so, how this might impact later fear conditioning.

Additionally, we measured USV emission on a single day between P8 and P10, which may have introduced additional variability in USV emission that could have impaired our ability to detect significant relationships between number of USVs emitted during infancy and the other outcome measures. We selected this age range based on past work that detected a significant relationship between number of USVs emitted and fear memory retention in later infancy (Harmon‐Jones et al. 2020), and all animals were randomly assigned to testing age. However, we cannot rule out the possibility that reducing variability in testing age might provide a more precise estimate of the relationship between separation‐induced USVs, fear memory retention during infancy, and learned and innate fear in adulthood.

## 5 Conclusions

The results of this study suggest that individual differences in infant rats’ retention of an aversive association predict an overall fearful phenotype in adulthood, although the significance of this relation was inconsistent across individual tasks. Importantly, we found that neither the sex of the animal nor exposure to an early life stressor (i.e., maternal separation) significantly impacted the size/direction of this relation. That is, rats that exhibited better memory retention during infancy were more likely to exhibit heightened anxiety‐like behavior in adulthood, regardless of their sex or prior exposure to early life adversity. These results suggest that individual differences in infants’ retention of an aversive association can be used to identify particularly high‐risk individuals within groups already known to be at increased risk of developing heightened anxiety. In addition, we did not find evidence that emitting a greater number of separation‐induced USVs during infancy predicts heightened fearfulness in adulthood. As such, it is likely more effective to assess individual differences in retention of an aversive association during infancy to identify and track animals that are prone to developing a fearful phenotype in adulthood than measuring number of separation‐induced USVs.

## Author Contributions

**Sylvia K. Harmon‐Jones**: conceptualization (equal), data curation (lead), formal analysis (lead), funding acquisition (supporting), investigation (lead), methodology (equal), visualization (lead), writing – original draft (lead), writing – review and editing (equal). **Rick Richardson**: conceptualization (equal), funding acquisition (lead), methodology (equal), supervision (lead), writing – review and editing (equal).

## Disclosure

An earlier version of the present manuscript was submitted as part of S.K.H.J.’s PhD thesis (Harmon‐Jones 2023).

## Conflicts of Interest

The authors declare no conflicts of interest.

## Supporting information




**Supplementary Materials**: dev70215‐sup‐0001‐SuppMat.docx

## Data Availability

The preregistration for this study is publicly available at osf.io/7yuwv, and the data that support the findings of this study are publicly available at osf.io/jmvfx/.
