# Supplementary material for: Heightened Fear Memory Retention During Infancy, but Not Number of Separation‐Induced Ultrasonic Vocalizations, Predicts a Fearful Phenotype in a Diverse Cohort of Rats
Source: Dev Psychobiol. 2026 Nov 3;68(6):e70215. doi: 10.1002/dev.70215 (PMC13731926; doi:10.1002/dev.70215)
Supplement: Supplementary file 1 [file DEV-68-e70215-s001.docx]

**Supplementary Results**

**Adulthood Performance – Effects of Proestrus**

There was no evidence of an effect of being in proestrus at the time of testing on performance on the pre-stress light-dark box (*t*_58_ = 0.29, *p* = .773, *d* = 0.09), the test of contextual fear (*t*_56_ = 0.65, *p* = .518, *d* = 0.20), or the post-stress light-dark box measure (*t*_56_ = 1.43, *p* = .159, *d* = 0.44). In addition, there was no evidence that being in proestrus at the time of contextual conditioning affected performance on the test of contextual fear (*t*_56_ = 0.69, *p* = .493, *d* = 0.21).

**Number of USVs in Infancy**

There was no significant effect of sex (*F*_1, 111_ = 0.03, *p* = .866; ηp^2^ = 0.00) nor interaction between sex and rearing (*F*_1, 111_ = 0.48, *p* = .488; ηp^2^ = 0.00) on number of USVs emitted. However, there was a significant effect of rearing (*F*_1, 111_ = 6.17, *p* = .015; ηp^2^ = 0.05) where maternally separated animals emitted more USVs than standard-reared animals.

**Infant Fear Memory Retention**

There was no significant effect of sex (*F*_1, 109_ = 1.12, *p* = .293, ηp^2^ = .01), rearing condition (*F*_1, 109_ = 1.80, *p* = .182, ηp^2^ = 0.02), nor their interaction (*F*_1, 109_ = 0.14, *p* = .707, ηp^2^ = 0.00) on infant memory retention.

**Pre-Stress Light-Dark Box in Adulthood**

There was a main effect of sex (*F*_1, 114_ = 32.26, *p* < .001, ηp^2^ = .221), where females exhibited more exploratory behavior (i.e., less innate fear) than males. However, there was no effect of early life adversity exposure (*F*_1, 114_ = 0.38, *p* = .537, ηp^2^ = 0.00), and there was no interaction between sex and exposure to early life adversity (*F*_1, 114_ = 1.51, *p* = .221, ηp^2^ = 0.01).

**Post-Stress Light-Dark Box in Adulthood**

There was a main effect of sex (*F*_1, 112_ = 32.76, *p* < .001, ηp^2^ = 0.23) where females exhibited more exploratory behavior (i.e., less innate fear) than males. However, there was no effect of early life adversity exposure (*F*_1, 112_ = 1.22, *p* = .273, ηp^2^ = 0.01) nor an interaction between early life adversity exposure and sex (*F*_1, 112_ = 2.55, *p* = .113, ηp^2^ = 0.02).

**Contextual Fear Conditioning in Adulthood**

There was a main effect of sex (*F*_1, 111_ = 8.58, *p* = .004, ηp^2^ = 0.07) where females had lower context-elicited freezing (*M* = 41.75, *SD* = 26.02) than males (*M* = 55.96, *SD* = 26.52). Again, there was no significant effect of early life adversity exposure (*F*_1, 111_ = 2.87, *p* = .093, ηp^2^ = .03) nor interaction between sex and exposure to early life adversity (*F*_1, 111_ = 0.02, *p* = .896, ηp^2^ = 0.00).

**Correlations between Measured Variables**

***Males***

Demonstrating better retention of an aversive association during infancy (i.e., higher CS-elicited freezing) predicted higher innate fear (i.e., reduced exploration) on the pre-stress light-dark box task, as predicted. While the other relations between retention of an aversive association during infancy and the adulthood measures (e.g., post-stress light-dark box and contextually-elicited freezing) were in the predicted direction, these relations did not reach statistical significance. Counter to prediction and Harmon-Jones et al. (2020), emitting more USVs was associated with poorer retention of an aversive association in infancy (as indexed by CS-elicited freezing).

Greater innate fear (i.e., reduced exploration) on the pre-stress light-dark box measure was associated with greater innate fear on the post-stress measure and heightened context-elicited fear. Greater innate fear on the post-stress light-dark box task was also associated with heightened context-elicited freezing (see Table S1).

***Females***

Demonstrating better retention of an aversive association during infancy (i.e., higher CS-elicited freezing) predicted higher context-elicited freezing in adulthood, as predicted. Opposite to prediction, there was a trend for animals that emitted a greater number of USVs to display lower innate fear (i.e., greater exploration) on the post-stress light-dark box measure (*p* = .073). None of the other correlations including the infant measures approached statistical significance.

Greater innate fear (i.e., reduced exploration) on the pre-stress light-dark box measure was significantly associated with greater innate fear on the post-stress measure, and there was a trend for greater innate fear on the pre-stress light-dark box measure to predict heightened context-elicited freezing (*p* = .077). Similarly, greater innate fear on the post-stress light-dark box measure was associated with higher context-elicited freezing (see Table S2).

**Table S1**

*Bivariate correlations between measured variables for male animals*

|  | Infant USVs | Infant CS-Elicited Freezing | Adult Pre-Stress  LDB | Adult Post-  Stress  LDB |
| --- | --- | --- | --- | --- |
| Infant CS-Elicited Freezing | -.29* |  |  |  |
| Adult Pre-Stress LDB | .12 | -.28* |  |  |
| Adult Post-Stress LDB | .08 | -.10 | .33* |  |
| Adult Context-Elicited Freezing | .02 | .22 | -.49*** | -.40** |

*Note.* USVs refers to ultrasonic vocalizations. LDB refers to Light-Dark Box. Adult Pre-Stress LDB and Adult Post-Stress LDB refer to the composite measures of LDB exploration from the pre-stress and post-stress tests. * *p* < .050, ** *p* < .010, *** *p* < .001.

**Table S2**

*Bivariate correlations between measured variables for female animals*

|  | Infant USVs | Infant CS-Elicited Freezing | Adult Pre-Stress  LDB | Adult Post-Stress  LDB |
| --- | --- | --- | --- | --- |
| Infant CS-Elicited Freezing | -.04 |  |  |  |
| Adult Pre-Stress LDB | .14 | -.03 |  |  |
| Adult Post-Stress LDB | .25# | -.19 | .55*** |  |
| Adult Context-Elicited Freezing | -.05 | .34* | -.24# | -.55*** |

*Note.* USVs refers to ultrasonic vocalizations. LDB refers to Light-Dark Box. Adult Pre-Stress LDB and Adult Post-Stress LDB refer to the composite measures of LDB exploration from the pre-stress and post-stress tests. # *p* < .090. * *p* < .050. ** *p* < .010. *** *p* < .001.

**Exploratory Factor Analysis**

**Table S3**

*Pattern matrix for the initial two-factor solution including all measured variables*

|  | Factor | | |
| --- | --- | --- | --- |
|  | 1 |  | 2 |
| Infant USVs | .116 |  | -.164 |
| Infant CS-Elicited Freezing | .093 |  | .**819** |
| Adult Pre-Stress LDB | **.684** |  | .073 |
| Adult Post-Stress LDB | **.891** |  | .069 |
| Adult Context-Elicited Freezing | **-.576** |  | .186 |

*Note.* Bolded factor loadings indicate that the measure loaded acceptably (i.e., > .50) on the respective factor. USVs refers to ultrasonic vocalizations. LDB refers to light-dark box. Adult Pre-Stress LDB and Adult Post-Stress LDB refer to the composite measures of LDB exploration from the pre-stress and post-stress tests.

**Table S4**

*Pattern matrix for the two-factor solution, excluding number of USVs*

|  | Factor | | |
| --- | --- | --- | --- |
|  | 1 |  | 2 |
| Infant CS-Elicited Freezing | .025 |  | .474 |
| Adult Pre-Stress LDB | **.710** |  | .061 |
| Adult Post-Stress LDB | **.837** |  | .039 |
| Adult Context-Elicited Freezing | **-.549** |  | .406 |

*Note.* Bolded factor loadings indicate that the measure loaded acceptably (i.e., > .50) on the respective factor. LDB refers to light-dark box. Adult Pre-Stress LDB and Adult Post-Stress LDB refer to the composite measures of LDB exploration from the pre-stress and post-stress tests.
